# GestaltMML: Enhancing Rare Genetic Disease Diagnosis through Multimodal Machine Learning Combining Facial Images and Clinical Texts

**Published:** 2024-04-22

**Authors:** Da Wu, Jingye Yang, Cong Liu, Tzung-Chien Hsieh, Elaine Marchi, Justin Blair, Peter Krawitz, Chunhua Weng, Wendy Chung, Gholson J. Lyon, Ian D. Krantz, Jennifer M. Kalish, Kai Wang

**Affiliations:** 1 Raymond G. Perelman Center for Cellular and Molecular Therapeutics, Children’s Hospital of Philadelphia, Philadelphia, PA 19104, USA; 2 Department of Biomedical Informatics, Columbia University Irving Medical Center, New York, NY 10032, USA; 3 Institute for Genomic Statistics and Bioinformatics, University Hospital Bonn, Rheinische Friedrich-Wilhelms-Universität Bonn, Bonn, Germany; 4 Department of Human Genetics, New York State Institute for Basic Research in Developmental Disabilities, Staten Island, NY, USA; 5 Division of Human Genetics, Children’s Hospital of Philadelphia, Philadelphia, PA 19104, USA; 6 Department of Pediatrics, Boston Children’s Hospital, Harvard Medical School, Boston, MA, USA; 7 Biology PhD Program, The Graduate Center, The City University of New York, New York, United States of America; 8 Department of Genetics, Perelman School of Medicine, University of Pennsylvania, Philadelphia, PA, United States; 9 Department of Pediatrics, Perelman School of Medicine, University of Pennsylvania, Philadelphia, PA, United States; 10 Department of Pathology and Laboratory Medicine, Perelman School of Medicine, University of Pennsylvania, Philadelphia, PA 19104, USA

**Keywords:** Multimodal Machine Learning, Artificial Intelligence, Large Language Models, Human Phenotype Ontology, Rare Genetic Disorders, Facial phenotyping

## Abstract

Individuals with suspected rare genetic disorders often undergo multiple clinical evaluations, imaging studies, laboratory tests and genetic tests, to find a possible answer over a prolonged period of time. Addressing this “diagnostic odyssey” thus has substantial clinical, psychosocial, and economic benefits. Many rare genetic diseases have distinctive facial features, which can be used by artificial intelligence algorithms to facilitate clinical diagnosis, in prioritizing candidate diseases to be further examined by lab tests or genetic assays, or in helping the phenotype-driven reinterpretation of genome/exome sequencing data. Existing methods using frontal facial photos were built on conventional Convolutional Neural Networks (CNNs), rely exclusively on facial images, and cannot capture non-facial phenotypic traits and demographic information essential for guiding accurate diagnoses. Here we introduce GestaltMML, a multimodal machine learning (MML) approach solely based on the Transformer architecture. It integrates facial images, demographic information (age, sex, ethnicity), and clinical notes (optionally, a list of Human Phenotype Ontology terms) to improve prediction accuracy. Furthermore, we also evaluated GestaltMML on a diverse range of datasets, including 528 diseases from the GestaltMatcher Database, several in-house datasets of Beckwith-Wiedemann syndrome (BWS, over-growth syndrome with distinct facial features), Sotos syndrome (overgrowth syndrome with overlapping features with BWS), NAA10-related neurodevelopmental syndrome, Cornelia de Lange syndrome (multiple malformation syndrome), and KBG syndrome (multiple malformation syndrome). Our results suggest that GestaltMML effectively incorporates multiple modalities of data, greatly narrowing candidate genetic diagnoses of rare diseases and may facilitate the reinterpretation of genome/exome sequencing data.

## INTRODUCTION

A substantial proportion of the global population, more than 6%, is affected by a rare genetic disorder^1^. While collectively common, rare diseases are individually rare^2^. Rare diseases are typically defined as affecting fewer than 200,000 people in the USA or less than one in 2,000 of the general population in Europe^3^. Based on the latest Orphanet^4^ and OMIM^5^ databases, currently there are at least 7000 rare genetic diseases. Due to the inherent rarity and extensive phenotypic heterogeneity of rare genetic disorders, accurately making a genetic diagnosis presents a challenge, often leading to a “diagnostic odyssey”^6–8^. Patients with suspected genetic syndromes often need to undergo multiple clinical evaluations, imaging studies, and laboratory tests, in addition to multiple modalities of genetic tests, including karyotype, chromosome microarray, gene panels, exome sequencing or genome sequencing, to make the diagnosis. Clinicians often encounter difficulties deciding what diagnostic test to use for efficient and accurate diagnosis, as they must navigate long differential diagnoses for each of many different symptoms. Shortening the odyssey could have significant clinical, psychosocial, and economic benefits^8,9^.

Many genetic diseases have distinctive facial features or dysmorphism (collectively considered the “facial gestalt”), which often provide important clues on facilitate diagnoses and expedite referrals to domain experts or suggest targeted genetic tests. In some cases, the recognition of a syndrome from a facial gestalt can be the first step in making a diagnosis^10^. However, the effectiveness of facial recognition relies heavily upon the clinician’s experience with facial recognition of syndromes. Given the many hundreds of rare genetic diseases with facial dysmorphisms, some only identified in the last 5 years, the facial recognition task is prohibitive for any clinician.

Following the recent success in Computer Vision (CV), there are several next generation phenotyping (NGP) approaches developed to analyze and predict rare genetic disorders based on patient’s 2D frontal facial images^11–13^. Among those, one widely known approach is called DeepGestalt^11^, which was developed by FDNA Inc. as the “Face2Gene” product, and was pretrained on deep convolutional neural network (DCNN) using CASIA^14^ and later fine-tuned on over 17106 patient frontal facial images with 216 disorders. However, DeepGestalt was only trained on a limited number of syndromes, which accounts for only a small proportion of the total syndromes. Adding a newly discovered syndrome requires collecting and adding new images and retraining the model. To make the model more inclusive for new “unseen” syndromes, GestaltMatcher^12^ was introduced as an improvement to DeepGestalt, in the sense that it takes the DeepGastalt’s feature layer before the final classification layer as a common embedding space (also known as “Clinical Face Phenotype Space” - CFPS) to encode learned facial dysmorphic features. Every frontal facial image was encoded into a 320-dimensional feature representation vector and by doing this, it can quantify the distance between different images and further identify the “closest match” among patients with known or unknown disorders, regardless of prevalence. Furthermore, one additional advantage of GestaltMatcher is that there is no need to alter the model’s architecture and retrain the model, when integrating newly identified syndromes. Despite these aforementioned successes, both DeepGastalt and GestaltMatcher use relatively dated model architecture and datasets for transfer learning introduced by Yi et al.^14^ More recently, Hustinx *et al.*^13^ updated the model architecture with more advanced iResNet^15^ and ArcFace^16^ and used various updated facial image datasets, including VGG2^17^, CASIA^14^, MS1MV2^16^, MS1MV3^16^ and Glint360K^18^, for pretraining. They also tried on different loss functions and proposed a model ensemble (combining three ArcFace models) to integrate face verification and disorder-specific models to improve performance on both seen and unseen syndromes. The model ensemble can achieve higher accuracy on unseen syndromes than all the previous models after fine-tuning.

Nonetheless, in numerous instances, facial images alone do not provide adequate information to make a precise diagnosis. For instance, syndromes such as Noonan syndrome (NS), Prader-Willi syndrome (PWS), Silver-Russell syndrome (SRS), and Aarskog-Scott syndrome (ASS) all have severe to moderate short stature^19^ which cannot be effectively reflected in frontal facial pictures. Additional phenotypic traits such as sleep disturbances, impaired balance and intellectual disability cannot be effectively captured by facial or other body photos. These aspects require additional data types (e.g., clinical notes). Moreover, numerous investigations^20–28^ have been conducted examining the contribution of age, sex, as well as racial and ethnic differences, to the phenotypic expression and frequency of various disorders and syndromes. Certain groups, often categorized as minorities, encounter challenges that stem from systemic biases ingrained within data availability, collection and analysis processes. These biases can inadvertently lead to misrepresentations, inaccuracies, and disparities in the rare genetic disorder predictions of these groups. Motivated by those facts, there are already some models developed trying to integrate facial images and clinical HPO terms together. The authors introduced the “prioritization of exome data by image analysis” (PEDIA) strategy^29^. PEDIA incorporates sequence variant interpretation with insights from the advanced phenotyping tool DeepGestalt. This approach enhances clinical assessments by combining expert human evaluation and artificial intelligence analysis, using frontal photographs to provide a more comprehensive assessment of individual clinical presentations. More recently, the PhenoScore^30^, an AI-based framework for analyzing genetic syndromes, was introduced and it comprises two modules: facial feature extraction from 2D photographs and HPO-based phenotypic similarity calculation. The framework uses a trained Support Vector Machine (SVM) for syndrome classification, based on extracted facial features and HPO similarities. However, these existing models process images and texts separately, then combine the results. This type of approach to integrating multi-modality data may lead to information loss, as it fails to fully capture the interaction between different modalities during training and it uses *ad hoc* methods to assign weight and combine information. In addition to the advancements mentioned earlier, a recent development in the field is DxGPT^31^, a text-only GPT-based model tailored for diagnosing rare genetic diseases. This model is built upon the closed-source GPT-4. In light of this, our objective is to create a multimodal machine learning (MML) methodology that incorporates a sophisticated modality interaction module. This methodology will handle both facial images and clinical texts in a uniform manner. The intended methodology aims to effectively merge patient facial images with textual information, which includes demographic details such as age, gender, and ethnicity, along with clinical notes, thereby preserving the integrity and richness of the data.

The recent progress in Transformer-based multimodal machine learning models has made our objective attainable. The story of Transformers started with the landmark paper “Attention is all you need”^32^ which introduces the so-called *self-attention mechanisms*, enabling the model to process sequence (e.g. texts sentences) in parallel rather than sequentially, like the traditional recurrent and convolutional neural networks. This design is revolutionary in the sense that it leads to improved performance, faster training, and scalability (larger size leads to better performance). Since then, Transformers have been extensively applied to both Natural Language Processing (NLP) and Computer Vision (CV), showcasing their versatility and effectiveness in various tasks. For instance, in NLP, Transformers have been applied to machine translation^33^, text generation^34^, sentiment analysis^35^, named entity recognition (NER)^36^, and others. In CV, tasks such as image classification^37^, object detection^38^, image segmentation^39^, image captioning^40^, visual question answering (VQA)^41^ now all rely on the Transformers. Recently, several multimodal machine learning models that leverage the strengths of Transformers have been developed, for instance, ViLT (Vision-and-Language Transformer Without Convolution or Region Supervision)^41^, CLIP (Contrastive Language–Image Pre-training)^42^, VisualBERT^43^, ALBEF (Align Before Fuse: Vision and Language Representation Learning with Momentum Distillation)^44^ and Google Gemini^45^.

Taking all these factors and tools into consideration, for the task of predicting rare genetic disorders, we introduce a novel methodology, *GestaltMML*, utilizing the ViLT (Vision-and- Language Transformer)^41^, which has the *simplest* design among vision-and-language models as it uses the nontrivial Transformer module for modality interaction learning while only using trivial (linear) vision and textual embedding.

## RESULTS

### Summary of the computational experiments

**Fig. 1.**
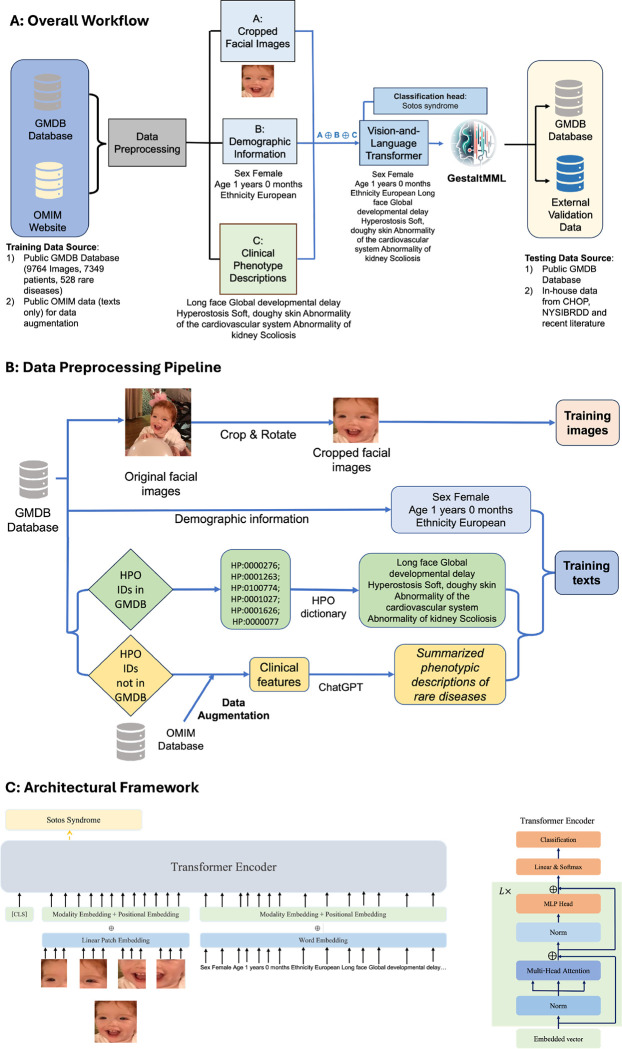
Overview of the GestaltMML. **A: Illustration of the overall workflow of the project.** GestaltMML uses information from facial images after appropriate pre-processing, demographic information as well as description of clinical phenotypes on each disease from both GMDB (if available) and the OMIM database. GMDB: GestaltMatcher Database. OMIM: Online Mendelian Inheritance in Man. CHOP: Children’s Hospital of Philadelphia. NYSIBRDD: New York State Institute for Basic Research in Developmental Disabilities. **B**: **Data Preprocessing Pipeline of GestaltMML, using Sotos syndrome as an example.** The facial images in GMDB were cropped by “FaceCropper” to crop and rotate the size of 112 * 112. The training texts can be divided into two categories: (1) Demographic information + HPO textual data, and (2) Demographic information + clinical features from OMIM database summarized by ChatGPT. **C: Architectural Framework of GestaltMML**: Based on the foundation of ViLT the structure of GestaltMML employs the Transformer encoder, capable of processing both textual and image inputs. Notice that this architecture closely resembles ViT, with the distinction that it solely accepts images as its input.

The overall workflow of the study is illustrated in Fig 1A. For GestaltMML, the data pre-processing procedure is illustrated in Fig 1B with an example encompassing both text and image data. The transformer model architecture is summarized in Fig 1C. In the following sections, we first demonstrate the performance of GestaltMML on the GestaltMatcher Database (GMDB)^12,46^. GestaltMML is a multimodal machine learning model that integrates facial images with demographic information and clinical HPO texts. GMDB is a collection of curated medical photographs of genetic syndromes as a resource for clinician and computer scientists. As shown in Table 1, the version of database (v1.0.9) that we used in this study contains 9764 frontal facial images from 7349 patients affected with 528 rare genetic disorders.

**Fig. 2.**
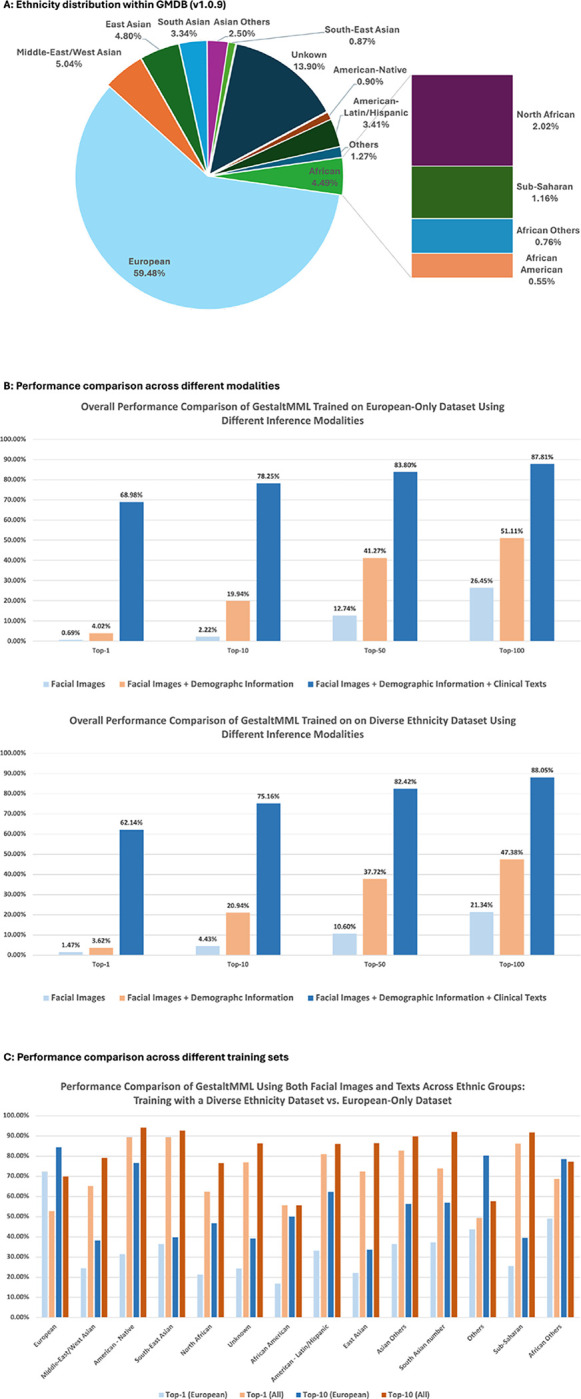
**A:** Ethnicity distribution in GMDB (v1.0.9). **B:** Overall accuracy by GestaltMML when using different modalities for inference. **C:** Comparative analysis of GestaltMML’s effectiveness trained on patients of European descents only vs. patients of all ethnic backgrounds. Both set of experiments use the same set of training and testing sets. The specifics regarding the sizes of the training and testing datasets are documented in **Table S3**.

The database includes patients of diverse ancestry through global collaboration. The specific ancestral categories represented are Middle-East/West Asian, American – Native, South-East Asian, North African, Unknown, African American, American - Latin/Hispanic, East Asian, Asian Others, South Asian, Others, Sub-Saharan, and African. Fig. 2A shows a significantly skewed distribution, with 59.48% of patients being of European ancestry. As highlighted in the recent efforts on constructing the diverse GMDB database ^46^, despite significant efforts to create as diverse a dataset as possible, achieving complete balance in patient characteristics is challenging due to the nature of rare diseases. This imbalance introduces inevitable difficulties for AI models in diagnosing rare genetic diseases, hence the decision to include ethnicity information in the textual data. **Fig. S1** illustrates a fairly equal distribution between males and females. There is an uneven age distribution, with a majority (64.90%) of patients being under 5 years old.

Following the convention in previous work on developing the ensembled image model^13^, for some evaluations, we measured performance on the GMDB-frequent and GMDB-rare subset, based on the number of patients for each disease; The GMDB-frequent contains disorders with more than (>) 6 patients, while GMDB-rare contains disorders with less or equal to (≤) 6 patients. Additionally, we will explore the significance of text and image features in GestaltMML and compare its effectiveness against current image-based models.

Finally, we extended our evaluation to several external validation datasets, encompassing patient data from the Children’s Hospital of Philadelphia (CHOP), New York State Institute for Basic Research in Developmental Disabilities (NYSIBRDD), and published literature. Our model also exhibits high performance on some of these external datasets, demonstrating the robustness of our methods.

### GestaltMML accurately classifies rare genetic diseases in GMDB

To thoroughly assess the predictive capabilities of the image-text pairs and overcome the challenge posed by a substantial amount of missing text data in cross-validation experiments, we constructed a train-test split, called *optimal train-test split*, in which the test set comprises images that meet two criteria: (1) they possess non-null present features and (2) they exclusively represent disorders that the training dataset has encountered with non-null features. For detailed algorithms for constructions of optimal train-test split with x:1 train: test ratio, see subsection “Construction of optimal train-test splits” in section “Methods.”

In the end, we constructed optimal train-test splits for each optimal train: test ratio from 1:1 to 9:1. The details on the number of images were summarized in **Table S1**. To make our results more robust, we constructed the optimal train-test splits using three different random seeds and calculated the means and standard deviations for all Top-1, Top-10, Top-50, Top-100 accuracies. Top-N accuracy is defined as the measure of precision when the actual disease label is included within the top-N predictions, whether from image information alone, or from the combined image and textual information. With optimal train: test ratio of 3:1, the model can reach mean accuracies of 72.54% for Top-1, 83.59% for Top-10, 88.96% for Top-50, and 91.64% for Top-100. The mean accuracies and their corresponding standard errors for other optimal train: test ratios are illustrated in **Table S2**.

By incorporating all the available images, demographic information, and clinical features, we observe that even with 3:1 train-test ratio, the model can reach high testing accuracy. While the accuracy is impressive, we acknowledge that the number of diseases that are analyzed is relatively small (528 in GMDB compared to the thousands of known rare diseases). Furthermore, there are likely ascertainment biases in typical facial image databases such as GMDB, such that only diseases for which there are characteristic dysmorphic features are documented, so the method may not work well for rare diseases without machine-recognizable facial features. Nevertheless, the use of demographic information and clinical phenotype information in GestaltMML can facilitate the prioritization of diseases in these cases.

### Feature importance analysis and comparisons with existing image models on GMDB dataset

Until now, almost all the existing literature has primarily centered on employing facial images alone for the prediction of rare genetic disorders. We selected the up-to-date ensembled image model documented in the previous work ^13^ as our initial benchmark model. We subsequently conducted an extensive comparative analysis. This included our modified versions of GestaltMML, aimed at exploring feature importance, discussed below. The summary of these comparisons can be found in Table 2. It is worth noting that GestaltMML *exclusively* employs the Transformer architecture and are completely devoid of convolution. In stark contrast, none of the other image-only models listed in previous work of ^13^ utilize the Transformer architecture. To make fair comparisons, we adopted the same train-test splits as in the previous work of developing ensembled image model^13^. Following the conventions in there^13^, we first separate the GMDB into GMDB-frequent and GMDB-rare. Recall that the GMDB-frequent contains disorders with more than 6 patients, while GMDB-rare contains disorders with less or equal to 6 patients. As described in Table 1 there are 8547 images from 6376 patients with 244 disorders in GMDB-frequent and 1217 images from 973 patients with 284 disorders in GMDB-rare. For GMDB-frequent, we used 7755 images for training and the remaining 792 images for testing. For GMDB-rare, we used the same 10-fold cross validation as in the previous work of developing ensembled image model^13^. On average, there are 856.9 images for training and the remaining 360.1 for testing. We fine-tuned the training data from combined GMDB-frequent and GMDB-rare and further evaluate on GMDB-frequent or GMDB-rare. Altogether, the training involves 8611.9 images, with 792 images designated for testing in GMDB-frequent and 360.1 images for testing in GMDB rare.

To delve deeper into the significance of each modality (images and texts) and understand their individual effects on the final prediction power, we conducted an evaluation procedure that we call “modality-masking.” To test the prediction power of images, we masked out all the text. We fine-tuned the training data with entire texts components replaced by “*” (facial image + *) on ViLT. We call the fine-tuned model GestaltViT to emphasize that during the fine-tuning process, we exclusively focus on fine-tuning the facial images and not the image-text pairs. Similarly, we assessed the predictive capabilities of text by excluding all images. Specifically, we fixed one patient photo for all training samples, while keeping all other aspects unchanged as described earlier. The results obtained from this test were solely evaluated on images containing existing HPO terms. The fine-tuned model is denoted as GestaltLT, with “LT” standing for “Language Transformer”. Noticeably, as shown in Table 2, GestaltViT exhibits poorer performance compared to the ensembled image model. This outcome is entirely expected due to the ViLT model’s linear patch embedding on the image parts and it aligns with the well-known *scaling property* of Transformer-based models, as discussed and compared in prior works like ^37^. It is reasonable to anticipate that with a larger training dataset, the performance of GestaltViT would further improve. Regarding GestaltLT, it exhibits only slightly inferior performance compared to GestaltMML and outperforms both GestaltViT and the ensembled image model. Despite the natural biases present in the textual data due to GMDB and the use of data augmentation via OMIM, it exhibits remarkable predictive capabilities.

### GestaltMML demonstrates enhanced diagnostic equity for patients from underrepresented groups

GestaltMML underwent training using the GMDB (v1.0.9), which encompasses data from several ethnically under-represented groups, identified as “Middle-East/West Asian,” “American – Native,” “South-East Asian,” “North African,” “Unknown,” “African American,” “American - Latin/Hispanic,” “East Asian,” “Asian Others,” “South Asian,” “Others,” “Sub-Saharan,” and “African.” In Fig. 2B, the mean accuracies of GestaltMML are showcased across various modalities used for inference. It is evident that clinical texts exert the most significant influence in enhancing performance, while demographic information also proves beneficial in augmenting results, particularly for minority patients. Fig. 2C illustrates that GestaltMML, by integrating frontal facial images, demographic details, and clinical texts, significantly enhances its predictive accuracy across under-represented ethnic groups, particularly when compared to training exclusively on individuals of European descent - with only rare exceptions. Further elaboration on the methodologies for dividing the training and testing sets, as well as the detailed training and testing processes, is provided in the Methods section. Complete results, encompassing Top-1, Top-10, Top-50, and Top-100 mean percentage accuracies for each individual ethnicity, along with their corresponding standard deviations, are documented in **Tables S4-S9**.

### GestaltMML performs well on external validation datasets

#### Overview

Although GestaltMML has demonstrated success on the GMDB database, we aim to evaluate its performance further on external validation data to access its resilience to potential bias inherent in the GMDB database. Several case studies, including Beckwith-Wiedemann syndrome (BWS), Sotos syndrome, NAA10 neurodevelopmental syndrome, Cornelia de Lange syndrome (CdLS) and KBG syndrome are described and discussed separately below.

In this section, we use GestaltMML trained on optimal training: test split with ratio 9:1 (**Table S1**). However, for NAA10 patients, we employed GestaltMML that was trained using GMDB v1.0.3, with an optimal train: test ratio of 4:1, to avoid train-test overlap (the NAA10 patients from external validation cohort were included in the recent database update). Furthermore, we also compare the performance of GestaltMML with the state-of-the-art ensembled image model developed in the previous work^13^.

#### Beckwith-Wiedemann Syndrome (BWS)

Beckwith-Wiedemann Syndrome (BWS) is one of the most common overgrowth syndromes^47–50^. It exhibits both genetic/epigenetic and clinical diversity^51,52^. BWS involves molecular aberrations within a cluster of imprinted genes on chromosome 11p15.5–11p15.4: Loss of methylation at imprinting control region 2 (IC2) on the maternal allele is found in about 50% of patients, while paternal uniparental isodisomy for the 11p15 region (pUPD11) occurs in about 20% of patients^53^. Key signs of overgrowth like macrosomia, organomegaly, and hemihypertrophy, which may not be adequately represented in frontal facial images^52,54,55^. Consequently, earlier image-based models may fall short in providing reliable diagnosis when these important clinical features are not considered. To address this limitation, a multimodal machine learning approach that incorporates clinical texts (in the form of HPO terms) is needed.

**Fig. 3.**
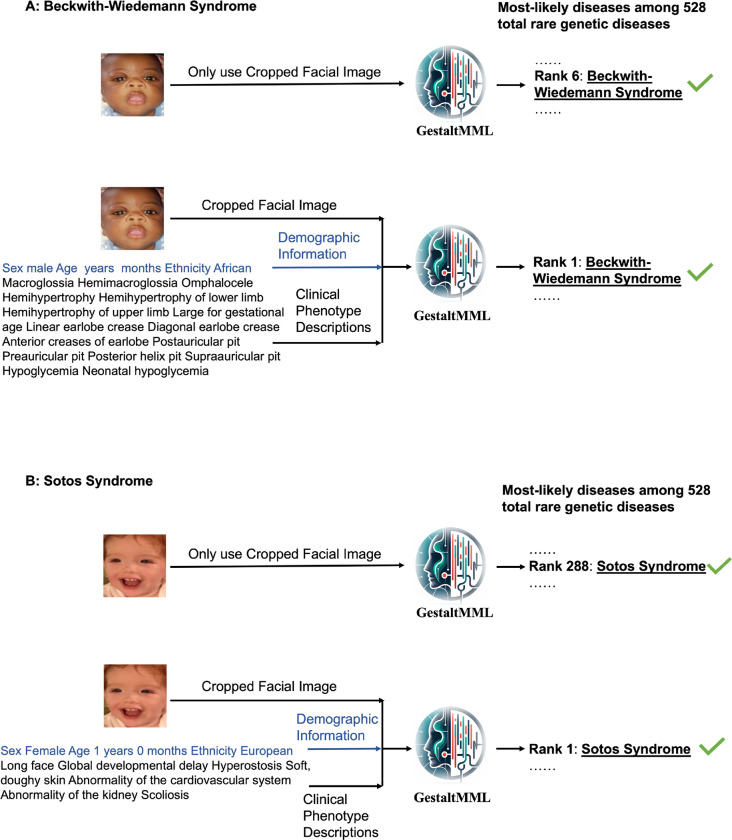
**Illustration demonstrating that multimodal inference, which combines facial images, demographic details, and clinical phenotype descriptions, is more effective than using facial images alone in diagnosing patients** with BWS (**A**) and Sotos Syndrome (**B**). The patient data for this study were sourced from CHOP with the appropriate consent.

We collected two groups of in-house patients affected with BWS at CHOP: one group with imprinting control region 2 loss of methylation (IC2) and another with paternal uniparental isodisomy of chromosome 11 (pUPD11). Each patient’s clinical phenotype is described, and all but one had complete demographic details, including sex, ethnicity, and age. We applied our GestaltMML to these data, with the findings presented in Table 3. The results show that clinical phenotypic descriptions are highly valuable, as GestaltMML achieves 100% detection Top-1 accuracy with this information. Nonetheless, the accuracy of GestaltMML markedly diminishes when it depends exclusively on facial images. Similarly, even the state-of-the-art ensembled image model struggles to accurately detect BWS. To illustrate, we showcase a particular example in Fig. 3A, where facial image information alone ranks BWS as the sixth most likely diagnosis yet adding demographic information and clinical phenotype descriptions improves the rank of the disease to the first.

#### Sotos Syndrome

Sotos syndrome is another rare genetic disorder characterized by excess growth during the early years of life, and children with Sotos syndrome typically have greater height, weight, and larger head size (macrocephaly) compared to their peers ^56–59^. Sotos syndrome frequently involves delays in motor skills, cognitive abilities, and social development. Since many of the clinical phenotypic features cannot be represented by facial photos, this syndrome is tested here to examine the importance of employing clinical texts for effective and accurate diagnosis, and to also compare with another overgrowth syndrome BWS.

We gathered data from 23 patients with Sotos Syndrome at the CHOP and conducted predictions using GestaltMML. The findings are detailed in Table 3, where again it is evident that incorporating multimodal data, including demographic details and clinical texts, greatly enhances the accuracy of inference. A particular instance of this is demonstrated in Fig. 3B, where facial image information alone ranks Sotos syndrome as the 288^th^ likely diagnosis yet adding demographic information and clinical phenotype descriptions improves the rank of the disease to the first. We also discovered that GestaltMML most frequently misdiagnoses Sotos syndrome as Marshall-Smith Syndrome (OMIM:602535), a genetic disorder characterized by distinctive facial traits such as prominent forehead, shallow orbits, blue sclerae, depressed nasal bridge, and micrognathia^60^.

#### NAA10-related Neurodevelopmental Syndrome

NAA10-related neurodevelopmental syndrome^61^ is an X-linked condition with a broad spectrum of findings ranging from a severe and often lethal phenotype cardiac in males (five deceased boys)^62^, to the severe NAA10-related intellectual disability in both males and females. In 2023, we expanded the phenotypic spectrum of NAA10-related neurodevelopmental syndromes through analysis of 56 individuals with NAA10 variants, demonstrating a phenotypic spectrum that includes variable intellectual disability, delayed milestones, autism spectrum disorder, craniofacial dysmorphology, cardiac anomalies, seizures, and visual abnormalities^63^. We collected clinical information (photos and clinical texts) on 68 subjects from NYSIBRDD. Note that they are not included in the previous version of the GMDB, i.e., v1.0.3, but most of them are now included in the new GMDB (v1.0.9). Therefore, results in this section are based on trained model on GMDB (v1.0.3) only.

**Figure 4.**
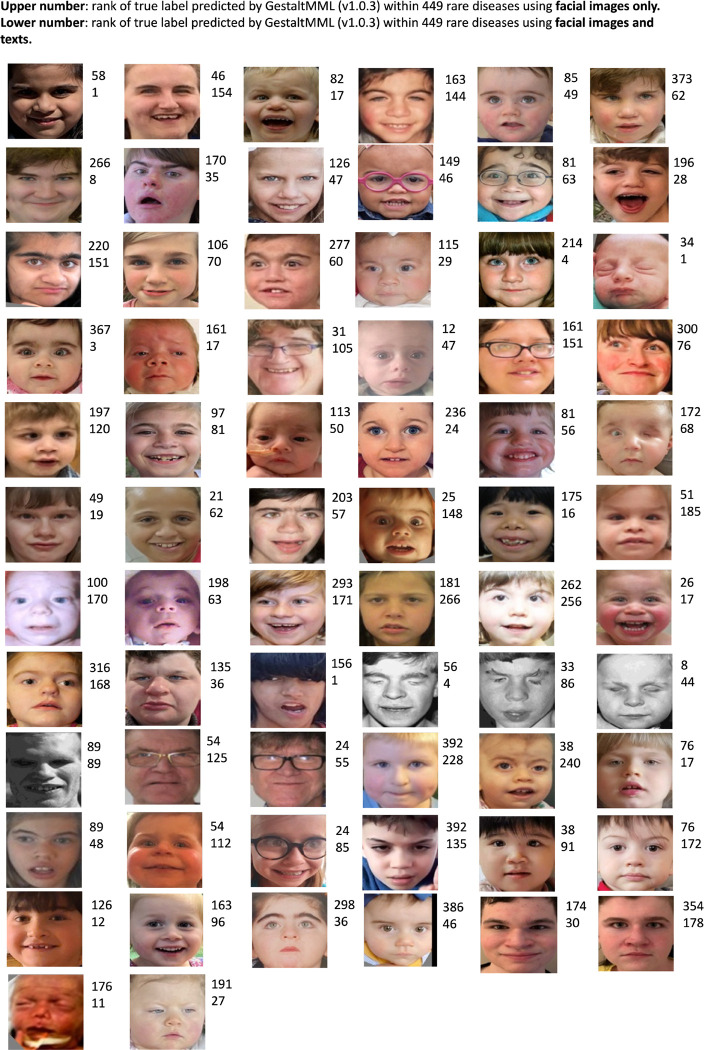
Rank of true label among 449 total disease labels for 68 NAA10 patients (with proper consent) from NYSIBRDD and recently published literature, as predicted by GestaltMML (trained on GMDB v1.0.3. with optimal train: test ratio of 4:1). The number on the upper and lower level indicate the ranking achieved using only facial images and using combined information (facial images, demographic data and clinical phenotype descriptions), respectively.

We used the same testing procedure as used earlier using GestaltMML. Regarding the text data, we extracted demographic information of patients and HPO terms from the clinical summaries provided by NYSIBRDD. Fig. 4 illustrates the ranking of true label among a total of 449 disease labels (total number of labels in GMDB v1.0.3), comparing results obtained from facial images alone with those derived from a combination of facial images, demographic information, and clinical phenotype descriptions. In almost all the cases, the use of multimodal information improves the prediction accuracy for GestaltMML significantly. In some instances, incorporating textual information can lead to poorer prediction outcomes. This primarily stems from the similarity of the text component in our test data to those of other neurodevelopmental disease labels in GMDB. Disease labels such as Intellectual Developmental Disorder, X-Linked, Syndromic 33 (OMIM:300966) and Developmental Delay, Hypotonia, Musculoskeletal Defects, and Behavioral Abnormalities (OMIM: 619595) have comparable textual descriptions. Such similarities can cause great confusion in the model and consequently degrade the results.

Additionally, we stress that demographic information can introduce certain biases in prediction outcomes. This bias is largely due to the uneven representation of demographic groups in the training set. For nearly all the diseases, most patients are under 5 years old and of European ancestry. When demographic information of a new patient falls outside the typical range in GMDB, the discrepancy is likely to result in unstable predictions. In future research, we intend to integrate more diverse data sources to improve the predictive power of GestaltMML, particularly for neurodevelopmental syndromes.

#### Cornelia de Lange syndrome (CdLS)

Cornelia de Lange syndrome (CdLS), also known as Brachmann-de Lange syndrome, is a genetically heterogeneous multiple malformation syndrome typically characterized by growth restriction, variable upper limb differences, hypertrichosis, long eyelashes, thick eyebrows, short nasal root and tip with anteverted nares, long philtrum and thin upper lip and other findings^64–66^.

The external validation dataset for CdLS patients, collected from CHOP with 19 samples, underwent the same evaluation through both GestaltMML and an ensemble image model, as detailed in Table 3. Contrary to previous conditions, CdLS diagnosis favored image-based over text-based analyses. We found that GestaltMML, even when utilizing only facial images, can surpass the performance of multimodal inference. Therefore, it is not unexpected that the ensembled image model can attain exceptionally high prediction accuracy. This is partially attributed to CdLS’s distinctive facial features that significantly aid in accurate diagnosis. However, textual data, while informative with details such as “global developmental delay” and “feeding difficulties,” tend to blur distinctions with other neurodevelopmental syndromes in GMDB (v1.0.9), impacting the model’s accuracy. Our analysis of this syndrome with well known facial features illustrate that GestaltMML may be more useful for other syndromes with subtle features that are hard to recognize by human experts.

#### KBG syndrome

KBG syndrome is an extremely rare, pan-ethnic, autosomal dominant disorder characterized by macrodontia, post-natal short stature, skeletal anomalies, abnormal hair implantation, and developmental delays^67–71^.

The external validation cohort for KBG syndrome from NYSIBRDD, comprising 18 samples, was evaluated using the same testing methodology. Results in Table 3 again indicated superior performance of image-based models over both multimodal and single-text models for KBG syndrome. Likewise, as with the previous case, this disparity is partly attributed to the older age of patients in the outside validation set compared to the training set, where encoding age as text introduced prediction instability. Additionally, same as the case of CdLS, the presence of common HPO terms in clinical texts, like “global developmental delay” and “intellectual disability, severe”, which overlap with many other rare diseases in GMDB (v1.0.9), contributed to predictions towards other neurodevelopmental syndromes despite the additional information provided by text data. This case study again highlighted the importance of having a training facial photo database with a large range of age distributions.

### GestaltMML exhibits outstanding performance in clustering diseases that have clinical similarities.

Finally, we performed a two-component UMAP clustering analysis on the logit values from the penultimate layer of the GestaltMML model (see Methods). This analysis focused on three comparative sets of diseases: BWS versus Sotos Syndrome, NAA10 versus NAA15-related syndromes, and KBG Syndrome versus Cornelia de Lange Syndrome (CdLS).

**Fig. 5.**
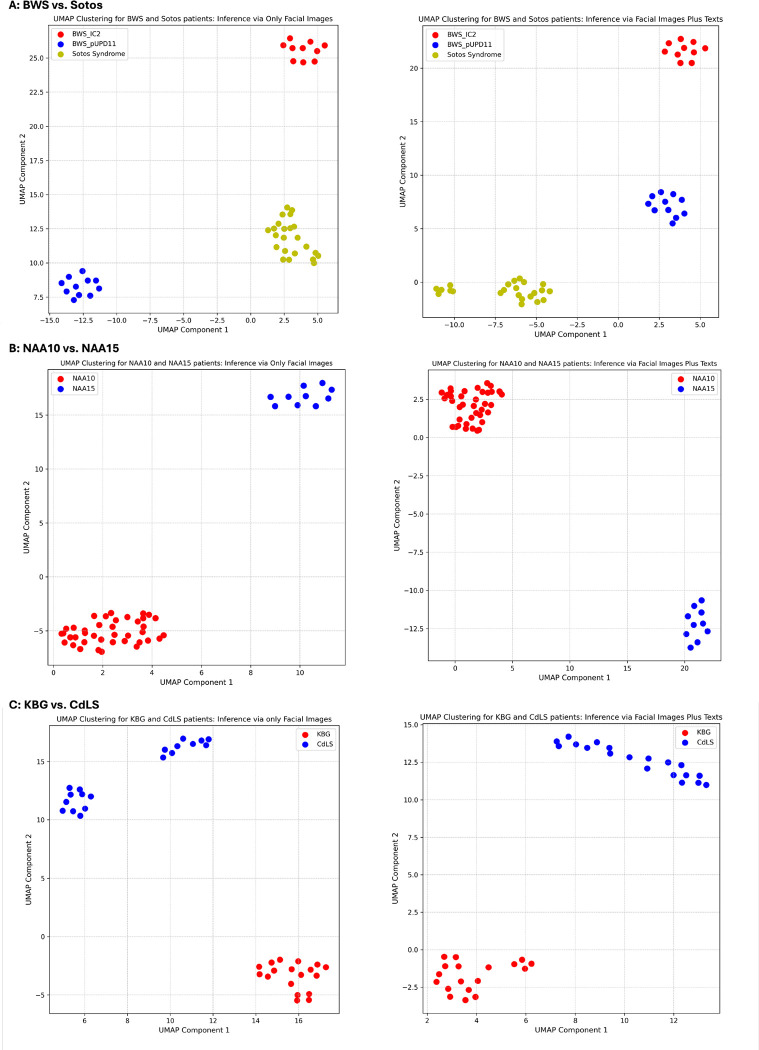
UMAP Clustering Analysis Across Three Comparative Sets. A: BWS Patient Cohorts (IC2 and pUPD11) Alongside Sotos Syndrome Patients B: NAA10 and NAA15 patients within GMDB (v1.0.9) C: KBG Syndrome and CdLS patients. For each comparison: On the left, inference is performed using only facial images; on the right, a multimodal approach combines facial images with textual data.

The first set of analysis focuses on external validation data including two BWS patient cohorts (IC2 and pUPD11, as previously discussed) in conjunction with patients with Sotos syndrome from previous section. While both diseases represent overgrowth syndromes, the model can clearly separate these two, and even separate the two genetic subtypes of the same syndrome (Fig. 5A).

Next, within the GMDB (v1.0.9), we evaluate GesltaltMML’s clustering efficiency for patients associated with NAA10 and NAA15-related neurodevelopmental syndromes (Fig. 5B). This analysis was confined to patients cataloged in GMDB (v1.0.9) only. We found that despite overlap of clinical phenotypes between these two syndromes, the model can still separate those affected with NAA10 deficiency versus NAA15 deficiency.

Lastly, we conducted tests using external validation data for patients with KBG syndrome and Cornelia de Lange syndrome (CdLS), as mentioned in the previous section (Fig. 5C). As expected, these two diseases can be separated. However, it is worth noting that among CdLS patients, facial image inference reveals two distinct clusters. Additional investigation found that this occurrence is attributed to background color variations: one cluster comprises images with white or pale backgrounds, while the other consists of images with warm or dark yellow backgrounds. This observation suggests that additional improvements to normalize background color may increase precision of the representation of facial images.

## DISCUSSION

In the current study, we introduced a novel multimodal approach, GestaltMML, to integrate frontal facial photos, clinical features, and demographic information together to narrow the differential diagnosis of rare genetic diseases. This approach is motivated by the observation that sole reliance on facial images of patients is insufficient to encompass all essential information required for the accurate diagnosis of rare genetic disorders. Our findings indicate that multimodal machine learning can lead to a substantial enhancement in the accuracy of predicting likely genetic diagnoses. Furthermore, it proves to be an indispensable resource for differentiating rare disorders with shared clinical characteristics via UMAP clustering analysis. Similar to the capabilities of the previously developed GestaltMatcher^12^ and the Ensembled image model^13^, this approach to clustering enables the model to automatically identify novel, previously unrecognized rare diseases without the need to alter the classification layers or undergo a complete retraining of the model. In combination with genome/exome sequencing data, GestaltMML is likely to greatly facilitate the interpretation or periodic reinterpretation of data, ultimately addressing the “diagnostic odyssey” challenge.

As previously mentioned, GestaltMML utilizes only the Transformer architecture. This choice aligns with the foundational principles outlined in the seminal paper “Attention is all you need,”^32^ which advocates for the complete substitution of recurrent or convolutional networks. Compared to classical CNN-based image models, additional technical differences and key innovations of our approach are discussed as follows: (1) Our methodology diverges from prior models that focused solely on facial images by incorporating both facial images and texts as inputs for the prediction of rare genetic disorders. This distinction sets it apart from the image-only models discussed in the previous work^13^ and the related references therein. (2) We integrated demographic data from patients, including sex, age, and race/ethnicity details, into the text inputs, enabling the model to discern distinct patterns for each rare disorder. We demonstrated that this approach successfully reduces biases inherent in data collection and analysis, especially regarding underrepresented minority groups, leading to a fairer diagnostic procedure. (3) We introduced a data augmentation technique leveraging the OMIM database^72^. This approach enhances the model’s training process by infusing it with a rich and comprehensive textual knowledge base. The incorporation of information from the OMIM database during training contributes to improved performance of the model. (4) We further examined the significance of textual and visual elements during multimodal training using *modality masking* techniques, offering valuable insights for future research endeavors. (5) Ultimately, upon evaluating GestaltMML and the ensembled image model across several external validation datasets, we observed a notable improvement in diagnostic accuracy for numerous conditions, such as BWS and Sotos syndrome, through the integration of textual information. Conversely, for diseases like CdLS and KBG syndrome, image-only models (this includes both the image segment of GestaltMML and the ensemble of image models) outperformed multimodal methods in terms of prediction effectiveness. These finding prompts clinicians to be judicious in using multimodal GestaltMML to assist in diagnosis, particularly when clinical HPO terms resemble those of many different disorders. The feature importance analysis of GestaltMML indicates that the text component’s predictive power exceeds that of images, suggesting that non-specific HPO texts may confuse the model and decrease its accuracy. Where facial images are distinctly recognizable, basing a diagnosis solely on these images might yield higher precision.

To optimize performance, the GestaltMML model leverages a straightforward approach of using concatenated HPO terms as textual inputs. Incorporating continuous clinical text paragraphs directly into these models may, however, affect the performance of GestaltMML adversely. The latest developments in large language models (LLMs) have significantly enhanced the capability to identify and extract HPO terms directly from clinical text paragraphs with high efficiency. A notable example is PhenoGPT^73^, which, built upon advanced large language models, demonstrates high accuracy in extracting HPO terms. Given its effectiveness, it is advisable to preprocess continuous clinical text paragraphs with PhenoGPT or similar large language models before feeding it into GestaltMML for optimal results.

Notwithstanding the several advantages and strengths outlined so far, it is important to acknowledge that our current GestaltMML methodology does have limitations. Here we highlight those limitations: (1) The major limitation pertains to image embedding within our GestaltMML framework. Our current approach employs linear patch embedding (same as ViT^37^), a method that has demonstrated comparably lower efficacy when compared to textual embedding, particularly when working with constrained training data. This limitation of the image modules also prompts caution regarding multimodal inference, particularly when text components lack distinctiveness, such as the cases of CdLS and KBG syndrome discussed before. To address this concern, we recommend the adoption of a more sophisticated feature extraction module, such as those based on Transformers or Convolutional Neural Networks (CNNs). For more comprehensive discussion, see literatures^42,43^. (2) The GestaltMML is constructed upon the foundations of ViLT, a pretraining framework designed for both images and text, albeit not specific to facial images with medical contexts. There is an intriguing avenue to explore: the creation of a foundational multimodal model that is pretrained using facial images (ideally from patients) alongside corresponding clinical textual descriptions. However, we are aware of the challenges to procure a large and diverse dataset encompassing patient facial images and medical captions, especially since much of the data are not in the public domain or are not consented for research use. Moreover, the training process for such a model might demand a substantial investment of time and computing resources. With the expansion of facial photo databases such as GMDB as well as the integration of photo information to clinical phenotype databases on rare diseases, it may be possible to create a foundational multimodal model down the road.

## METHODS

### Patients and Photos

The study to develop multimodal machine-learning approaches for rare disease diagnosis was approved by the Institutional Review Board of the Children’s Hospital of Philadelphia (IRB 18–015712). The GMDB (v1.0.9) database used in the current study contains 9764 images from 7349 patients affected with 528 genetic disorders, obtained from https://db.gestaltmatcher.org/. BWS and Sotos syndrome images were collected and analyzed under the oversight of the Children’s Hospital of Philadelphia (CHOP) Institutional Review Board protocol (IRB 13–010658). The collection and analysis of data on individuals with Cornelia de Lange syndrome were performed under CHOP Institutional Review Board protocol (IRB 16–013231). In brief, consent was obtained from all patients and/or legal guardians to analyze and in some case publish the images. For collection and facial phenotyping analysis on the NAA10-related neurodevelopment syndrome and KBG syndrome, both oral and written patient consent were obtained for research and publication, with approval of protocol #7659 for the Jervis Clinic by the New York State Psychiatric Institute - Columbia University Department of Psychiatry Institutional Review Board. Written family consent was given for publication of any photographs of the children.

### Training and Evaluation of GestaltMML

#### Overview of training data sources

In the developments of GestaltMML, we will primarily use two sources of data. The first one is the GMDB database, which contains frontal facial images of patients and corresponding textual metadata. It is open to researchers in medical domains, and one needs to apply for access first to use the data. The second one is the OMIM website^72^ which serves as our ground knowledge base for rare genetic disorders. To deal with large amounts of missing textual data in GMDB, we will use the textual data from OMIM database for the purpose of data augmentation. The visual representation of whole data preprocessing procedure can be found in Fig. 1B.

#### Image data preprocessing

Our training and test images were cropped by the open source “FaceCropper” described in the GitHub page ^13^. In our experiment, the facial images are of dimension 112 * 112. Alternatively, the image can be manually resized to these dimensions, ensuring that the primary face encompasses the entire picture. Notice that the original facial images are subjected solely to cropping, without any alterations such as flipping, rotating, or converting to grayscale.

#### Text data preprocessing

To make the textual data preprocessing procedure clearer, we will separate our discussions into two cases.

The first case concerns images that have non-null present features, or equivalently, at least one HPO id in the “present features” column of the metadata. In this case, we will do the following two steps: (1) Transform the HPO id(s) into real text data via the standard HPO dictionary^74^ and then concatenate them with empty space in between. For instance, the “HP:0000486; HP:0001263; HP:0010864” will become “Strabismus Global developmental delay Intellectual disability severe.” (2) Add patients’ demographic information in the front. The image metadata of GMDB database contains patients’ sex, age, and ethnicity (or ethnicity note), which will be combined for our model training. For instance, the demographic textual data will look like “Sex male Age 4 years 8 months Ethnicity European.” If there is missing information, then we will simply leave that space empty. Therefore, the combined textual data in our first case will look like “Sex male Age 4 years 8 months Ethnicity European Strabismus Global developmental delay Intellectual disability severe.”

The second case deals with the case when images do not have present features at all, or equivalently, no HPO id in the “present features” column of the metadata. In this case, we will do the following two steps: (1) Use the textual data in the “clinical features” section of OMIM database as the primary source for data augmentation. Due to the limitations of model inputs’ length and for the sake of saving computing power and budgets, we further use OpenAI’s ChatGPT^75^ to summarize those texts within 500 tokens. The prompt we gave is “Summarize most crucial phenotype characteristics of the following texts describing clinical features of some rare genetic disorder within 500 tokens”. The sample texts paragraph (after summarization by ChatGPT) looks like “Clinical features of this rare genetic disorder include supravalvular aortic stenosis SVAS mental retardation distinctive facial features dental anomalies peripheral pulmonary artery stenosis infantile hypercalcemia statural deficiency characteristic dental malformation and a hoarse voice Other features may include renal abnormalities cardiovascular disease joint limitations hypotonia delayed growth cataracts stroke and cognitive deficits Patients often have musical and verbal abilities but struggle with visual-motor integration and attention deficit disorder They may also exhibit hypersensitivity to sounds and have urinary abnormalities.” Note that we also remove all the “,”, “.”, “:”, “()”, etc. to save token space for training. Same thing applies to the first case. (2) Likewise, add patients’ demographic information in the front.

The text data pre-processing approach mentioned is tailored exclusively for the GMDB database. Like mentioned before, for clinical practitioners working in real-world settings, it is recommended to employ PhenoGPT for the extraction of HPO terms from clinical text paragraphs. This should be followed by concatenating these terms with demographic data, as previously described, to ensure optimal data preparation and analysis.

#### Construction of optimal train-test splits

The optimal x:1 split in **Table S1** is constructed as follows: (1) Select all the disorders that have images with non-null present features and denote the set of such disorders D. (2) For each disorder d in D, let $I_{d}$ denote the set of all the image ids of disorder d with non-null present features and compute the cardinality $\left|I_{d}\right|$. Next, randomly select $\left[\left|I_{d}\right|/(x+1)\right]$ image ids from $I_{d}$ and call them $I_{d}^{t}$. For instance, if $\left|I_{d}\right|=5$ and train:test ratio is 3:1, then [5/(3+1)] = 1 image will go to the test set $I_{d}^{t}$ and rest 4 images will be grouped into training set. On the other hand, if $\left|I_{d}\right|=2$ and train:test ratio is 3:1, then [2/(3+1)] = 0 image will go to test set. In other words, we do not select any test image under this optimal train-test split ratio. (3) The total testing set is simply the union ${⋃}_{d\in D}\;\;I_{d}^{t}$ and the training set is the complement of the testing set.

Using the above algorithm, we constructed optimal train-test splits for each optimal train: test ratio from 1:1 to 9:1. To make our results more robust, we will repeat the above algorithms three times (using three different random seeds) and calculate the means (**Table S1**) and standard deviations (**Table S2**) for all Top-1, Top-10, Top-50 and Top-100 accuracies.

#### Train-test splits for experiments of GestaltMML enhancing diagnostic equity for patients from minority groups.

In Fig. 2B, we assess the effect of only including demographic information and including both demographic information and clinical HPO terms in the textual component. The text component will look like (1) “*” (no texts at all), (2) “Sex male Age 4 years 8 months Ethnicity European” (demographic information only) or (3) “Sex male Age 4 years 8 months Ethnicity European Strabismus Global developmental delay Intellectual disability severe” (demographic information and clinical HPO texts).

In Fig. 2C, we evaluate the diversity of training datasets that include both facial images and textual information. We begin with an optimal training to testing ratio of 4:1 (from previous section), using three different random seeds, and exclusively use patients of European descent for the entire training set. For comparison, we then include patients from all ethnic backgrounds in the training set. We use the same three seeds for the optimal train: test ratio of 4:1. To keep the training set size comparable, we reduce the number of white patients by 72% for each disease. In both scenarios, we limit the testing dataset to include only patients whose diagnostic diseases are already present in the training set. The results for the top-1 and top-10 accuracies are shown in Fig. 2C, labeled as “Top-1 (European)” and “Top-10 (European),” respectively.

Complete results, including Top-1, Top-10, Top-50, and Top-100 mean percentage accuracies for each individual ethnicity, along with their corresponding standard deviations, are documented in **Tables S4-S9**.

#### Training and testing of GestaltMML

We fine-tuned our training set on ViLT (see Fig. 1) and then tested on the various test sets. The testing data may include both data from GMDB and external validation data. The Vision-and- Language Transformer (ViLT)^41^ utilized transformer encoder as modality interaction module and was pretrained on four datasets: Microsoft COCO (MSCOCO)^76^, Visual Genome (VG)^77^, SBU Captions (SBU)^78^, and Google Conceptual Captions (GCC)^79^. The statistics of these four datasets were reported in Table 1 of the original paper^41^*.*

#### UMAP Clustering Analysis of GestaltMML.

In Fig. 4, showcasing the UMAP clustering outcomes, we employed the logit values derived from the GesaltMML’s penultimate layer (immediately preceding the final softmax layer), resulting in a matrix of dimensions n × 528, where “n” represents the total number of test samples. For the UMAP fitting on this n × 528 matrix, composed of stacked logit values, we configured the parameters as follows: n_neighbors = 7, min_dist = 0.1, and n_components = 2.

For UMAP clustering of external validation data (Fig. 4A and Fig. 4C), we used GestaltMML trained with an optimal train-to-test ratio of 9:1. For the comparison between NAA10 and NAA15 (Fig. 4B), to ensure a sufficient number of samples in the test set, we selected GestaltMML with an optimal train-to-test ratio of 4:1.

## Figures and Tables

**Table 1.** Overview of the GMDB (v1.0.9) dataset, including the GMDB-frequent subset and the GMDB-rare subset. The GMDB-frequent contains disorders with more than (>) 6 patients, while the GMDB-rare contains disorders with less or equal to (≤) 6 patients.

Dataset	# of images	# of patients	# of disorders	# of images with clinical HPO texts
**GMDB-frequent**	8547	6376	244	3962
**GMDB-rare**	1217	973	284	470
**Total**	9764	7349	528	4432

**Table 2.** Performance of GestaltMML, GestaltViT and GestaltLT and their comparisons with that of baseline Ensembled image model in ^13^: All the models have undergone fine-tuning on the GMDB (v1.0.9). The models with underline indicates that we *exclusively* tested them on a subset of the total test set containing the available test modalities.

Model	GMDB-frequent		GMDB-rare	
	Top-1	Top-10	Top-1	Top-10
**GestaltMML**	50.14%	75.50%	24.14%	41.38%
**GestaltLT**	46.40%	74.93%	24.16%	41.38%
**GestaltViT**	18.16%	44.67%	6.97%	18.12%
**Ensembled image model**	43.02%	72.44%	19.77%	38.57%

**Table 3.** Results of GestaltMML on patients diagnosed with Beckwith-Wiedemann Syndrome (BWS), Sotos Syndrome and Cornelia de Lange Syndrome (CdLS) at the Children’s Hospital of Philadelphia, and KBG syndrome at the New York State Institute for Basic Research in Developmental Disabilities and recently published literature.

Beckwith-Wiedemann Syndrome (BWS)								
Model	Cohort (subset)	Testing Modalities	Percentage Accuracy				Sample Size (outside validation)	# of images in GMDB (v1.0.9)
			Top-1	Top-10	Top-50	Top-100		
GestaltMML	IC2	Images + Texts	100%	100%	100%	100%	10	26
GestaltMML	IC2	Texts only	90%	100%	100%	100%	10	26
GestaltMML	IC2	Images only	10%	40%	70%	90%	10	26
Ensembled image model	IC2	Images only	20%	60%	90%	100%	10	26
GestaltMML	pUPD 11	Images + Texts	100%	100%	100%	100%	11	26
GestaltMML	pUPD 11	Texts only	100%	100%	100%	100%	11	26
GestaltMML	pUPD 11	Images only	0%	27.27%	45.45%	54.54%	11	26
Ensemble d image model	pUPD 11	Images only	8.33%	66.67%	91.67%	100%	11	26

Sotos Syndrome							
Model	Testing Modalities	Percentage Accuracy				Sample Size (outside validation)	# of images in GMDB (v1.0.9)
		Top-1	Top-10	Top-50	Top-100		
GestaltMML	Images + Texts	73.91%	86.96%	95.65%	95.65%	23	126
GestaltMML	Texts only	69.57%	82.61%	95.65%	95.65%	23	126
GestaltMML	Images only	0%	4.34%	17.39%	30.43%	23	126
Ensembled image model	Images only	41.93%	61.29%	93.55%	95.16%	23	126
NAA10-related Neurodevelopmental Syndrome							
Model	Testing Modalities	Percentage Accuracy				Sample Size (outside validation)	# of images in GMDB (v1.0.3)
		Top-1	Top-10	Top-50	Top-100		
GestaltMML	Images + Texts	4.41%	32.35%	66.18 %	82.35%	68	15
GestaltMML	Texts only	5.88%	38.23%	72.06%	82.35%	68	15
GestaltMML	Images only	0.00%	1.47%	23.53%	41.18%	68	15
Ensembled image model	Images only	7.35%	16.18%	44.12%	66.18%	68	15
Cornelia de Lange Syndrome (CdLS)							
Model	Testing Modalities	Percentage Accuracy				Sample Size (outside validatio n)	# of images in GMDB (v1.0.9)
		Top-1	Top-10	Top-50	Top-100		
GestaltMML	Images + Texts	21.05%	73.68%	84.21%	94.74%	19	382
GestaltMML	Texts only	0.00%	47.36%	84.21%	89.47%	19	382
GestaltMML	Images only	52.63%	68.42%	84.21%	94.74%	19	382
Ensembled image model	Images only	76.67%	86.67%	100%	100%	19	382
KBG Syndrome							
Model	Testing Modalities	Percentage Accuracy				Sample Size (outside validation)	# of images in GMDB (v1.0.9)
		Top-1	Top-10	Top-50	Top-100		
GestaltMML	Images + Texts	44.44%	83.33%	88.89%	88.89%	18	167
GestaltMML	Texts only	38.89%	72.22%	88.89%	88.89%	18	167
GestaltMML	Images only	55.56%	66.67%	83.33%	88.89%	18	167
Ensembled image model	Images only	94.44%	94.44%	100%	100%	18	167

## Data Availability

The GMDB (v1.0.9) database used in the current study can be obtained from https://db.gestaltmatcher.org/. All the software tools and computational workflow (as Jupyter Notebook) can be found at https://github.com/WGLab/GestaltMML. This study did not generate any new material.
